# From embolization to access: Wire-enabled crossing of a coil pack for targeted distal re-treatment

**DOI:** 10.1016/j.radcr.2026.07.050

**Published:** 2026-08-19

**Authors:** Justin Lee, Daniella Boros, Edward W. Lee, Frank Hao

**Affiliations:** a Department of Radiology, Division of Interventional Radiology, UCLA Medical Center, Los Angeles, CA, USA; b Department of Surgery, Division of Liver and Pancreas Transplantation Surgery, UCLA Medical Center, Los Angeles, CA, USA

**Keywords:** Interventional, Radiology, Embolization, Access

## Abstract

This brief report describes a unique case in which distal splenic embolization was achieved by traversing a previously placed coil pack in the proximal splenic artery. Following orthotopic liver transplantation, A 65-year-old woman developed poor hepatic arterial flow which was initially managed by proximal splenic artery coil embolization to improve hepatic perfusion. Her postoperative course also notable for persistent thrombocytopenia and splenomegaly. Distal splenic access was obtained by crossing the previously placed coil pack with a hydrophilic wire which enabled successful distal administration of liquid embolic. This report highlights the feasibility of crossing a coil pack with a wire to facilitate targeted re-embolization.

## Introduction

Splenic artery embolization is commonly used to modulate hepatic perfusion and augment arterial inflow to the allograft in liver transplant recipients [1]. Although proximal coil embolization is often effective, persistent splenomegaly and thrombocytopenia may indicate inadequate reduction in splenic perfusion or reconstitution via collateral formation [2,3]. Repeat intervention can be technically challenging when a dense, proximal coil pack prevents conventional catheterization of the distal splenic artery. We describe, to our knowledge, the first reported case of wire transversal through a previously deployed splenic artery coil pack to regain distal arterial access and permit targeted delivery of liquid embolic material.

## Case report

**Fig. 1:**
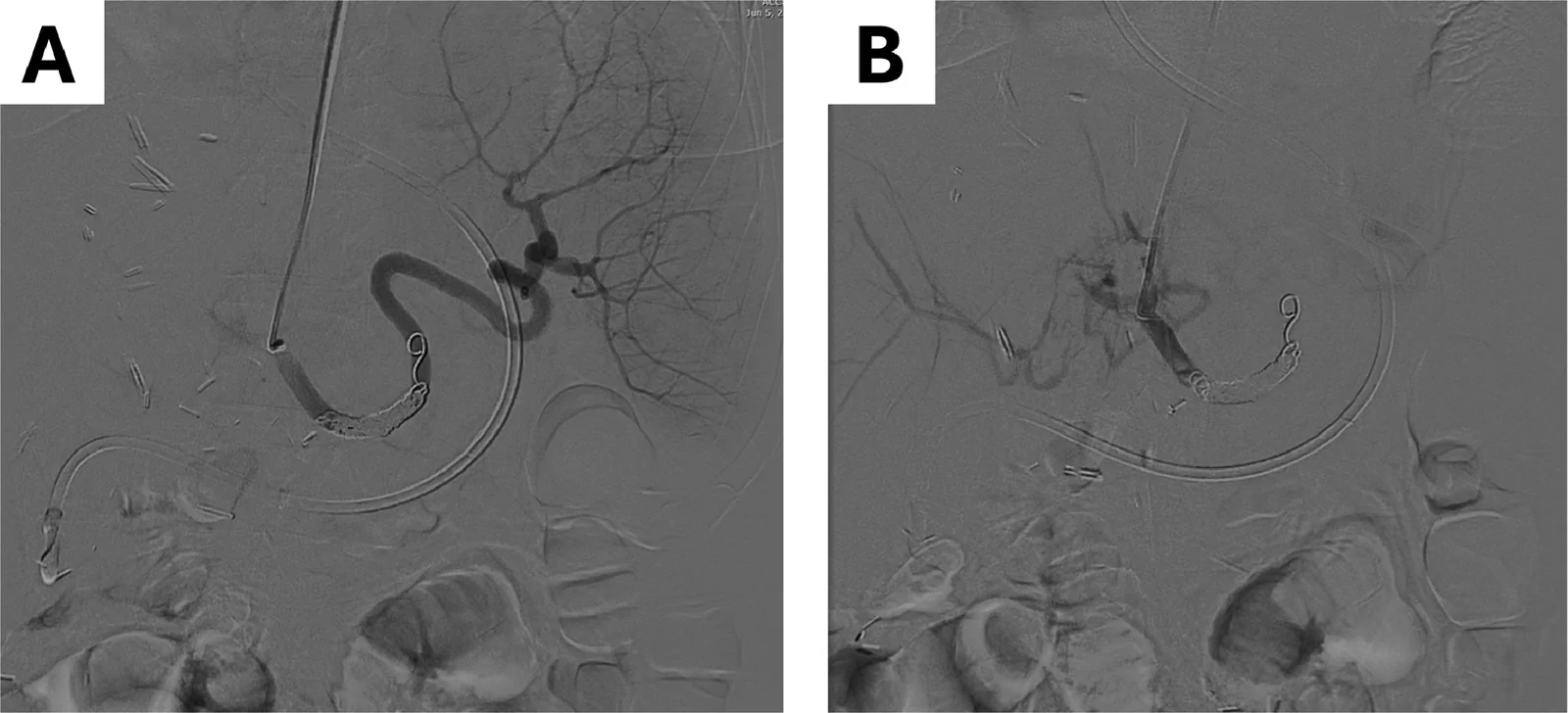
Initial coil embolization (A) with application of Gelfoam slurry to flow stasis (B).

This single-institution retrospective report was exempt from institutional review board approval. A 65-year-old female patient with a remote history of iatrogenic biliary injury during open cholecystectomy with resultant multiple biliary reconstructions leading to cirrhosis with decompensation presented with hepatic dysfunction following orthotopic liver transplantation (OLT). The patient’s postoperative course was complicated by poor hepatic arterial flow, resulting in ischemia of the allograft and thrombocytopenia in the setting of splenomegaly. A proximal splenic artery embolization was performed initially to help achieve a hepatic artery buffer response, where a dense coil pack was formed between the pancreatic magna and dorsal pancreatic arteries using 4 Terumo Azur Cx coils: 6 mm × 17 cm, ×3 and 8 mm × 24 cm, ×1 (Terumo Interventional Systems, Aliso Viejo, CA) (Fig. 1A). Following application of Gelfoam (Pfizer, New York, NY) slurry into the coil pack, a subsequent splenic artery angiogram demonstrated complete stasis (Fig. 1B).

Two months after the proximal splenic artery embolization, the patient's severe thrombocytopenia persisted. A trial of dexamethasone and IVIG did not improve platelet counts. At this point, a multidisciplinary decision was made to attempt distal splenic artery embolization to address potential splenic sequestration. Given the presence of the dense proximal splenic artery coil pack, direct splenic arterial access and navigation of collateral vasculature were considered.

**Fig. 2:**
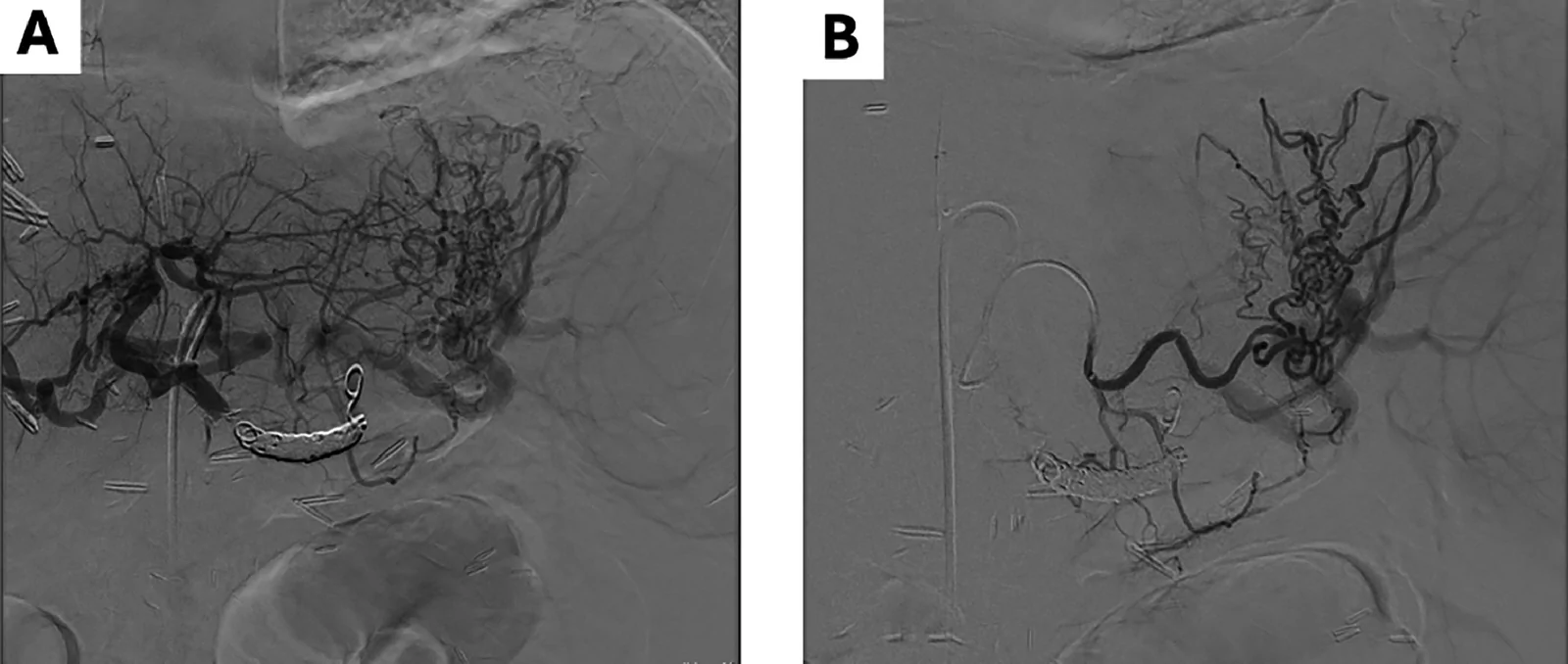
(A) Splenic artery angiogram 3 months after initial coil embolization demonstrating significant collateral formation with distal artery reconstitution and (B) attempt to obtain distal splenic artery access through the collateral system.

**Fig. 3:**
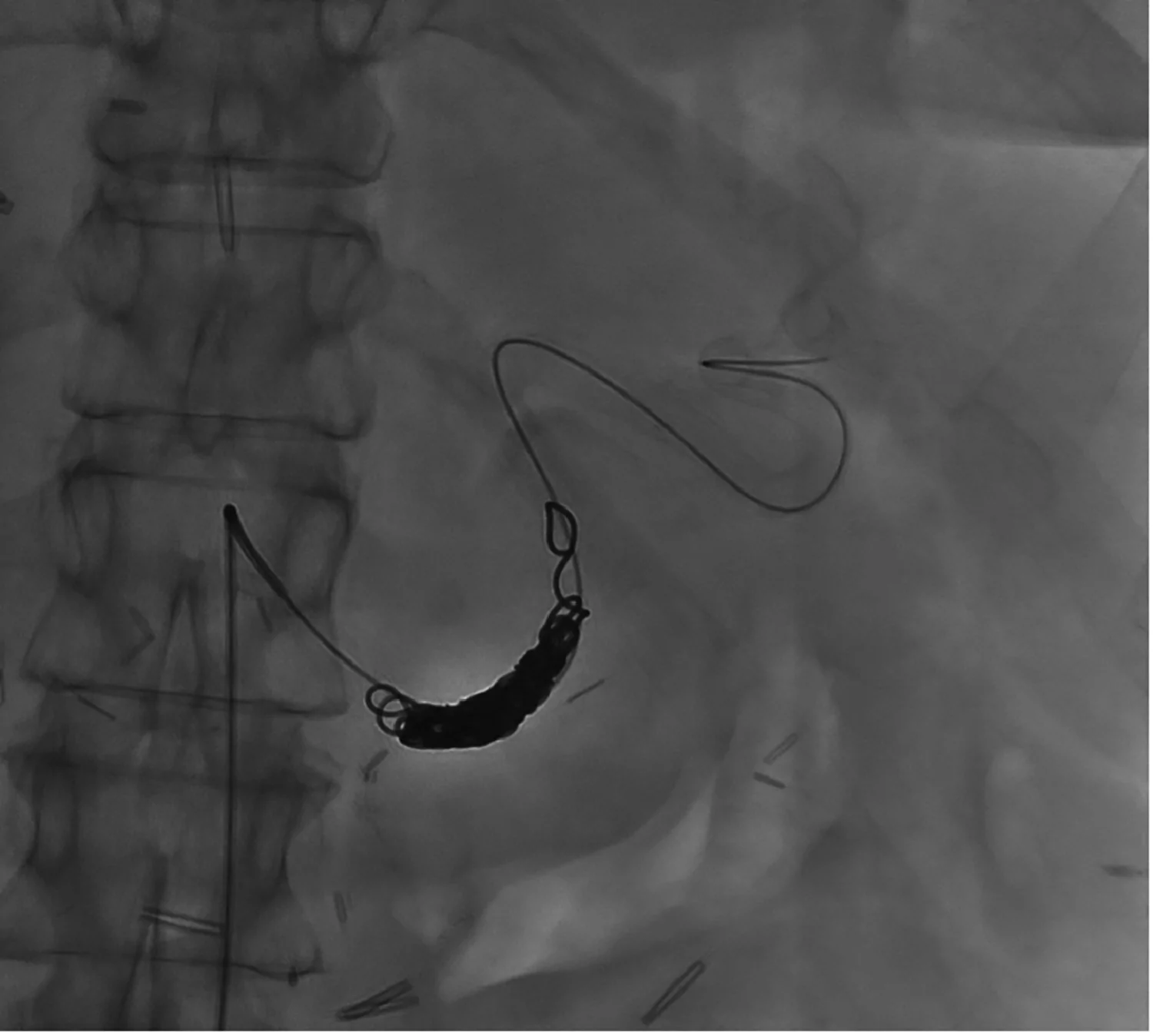
Successful direct recanalization through the coil pack using an 035 Glidewire.

**Fig. 4:**
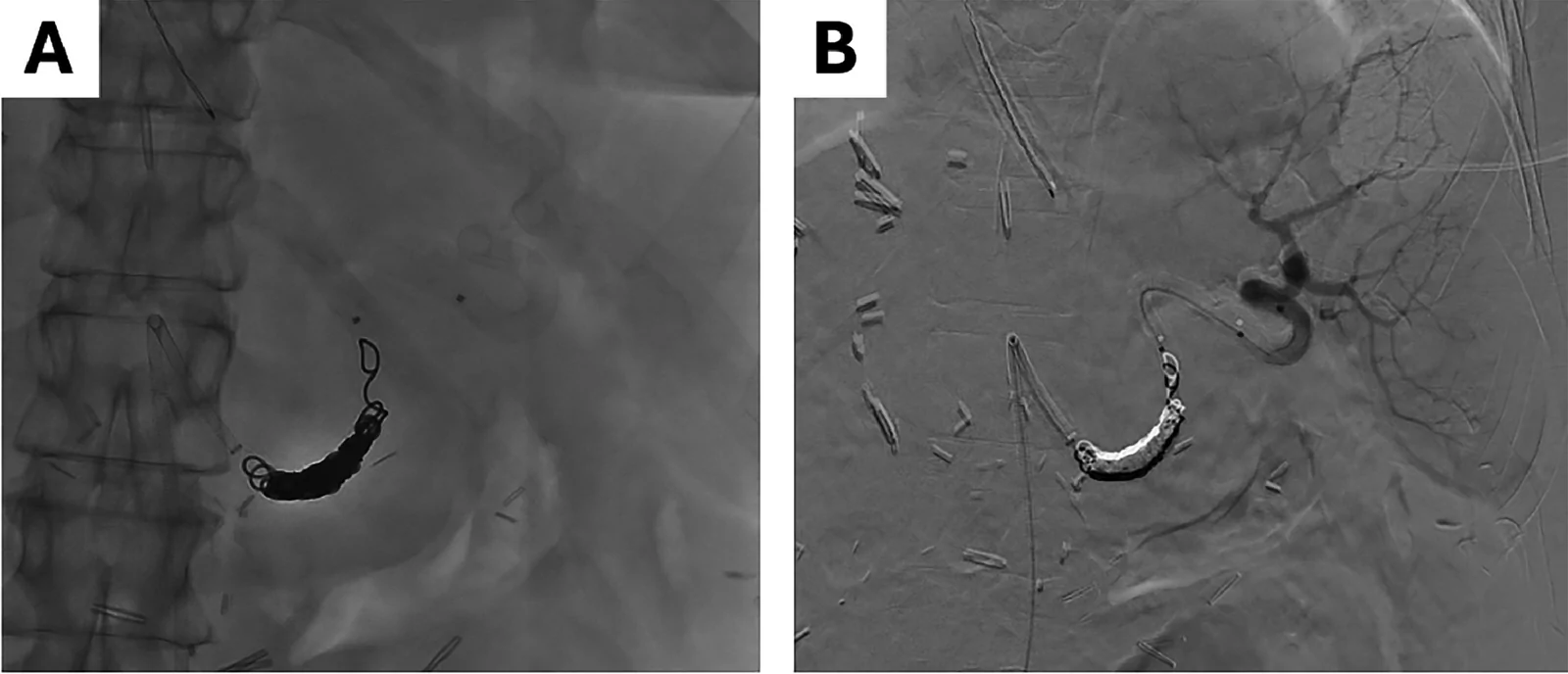
Navigation through the coil pack with a 5Fr CXI catheter (A) with successful distal splenic artery angiography (B).

**Fig. 5:**
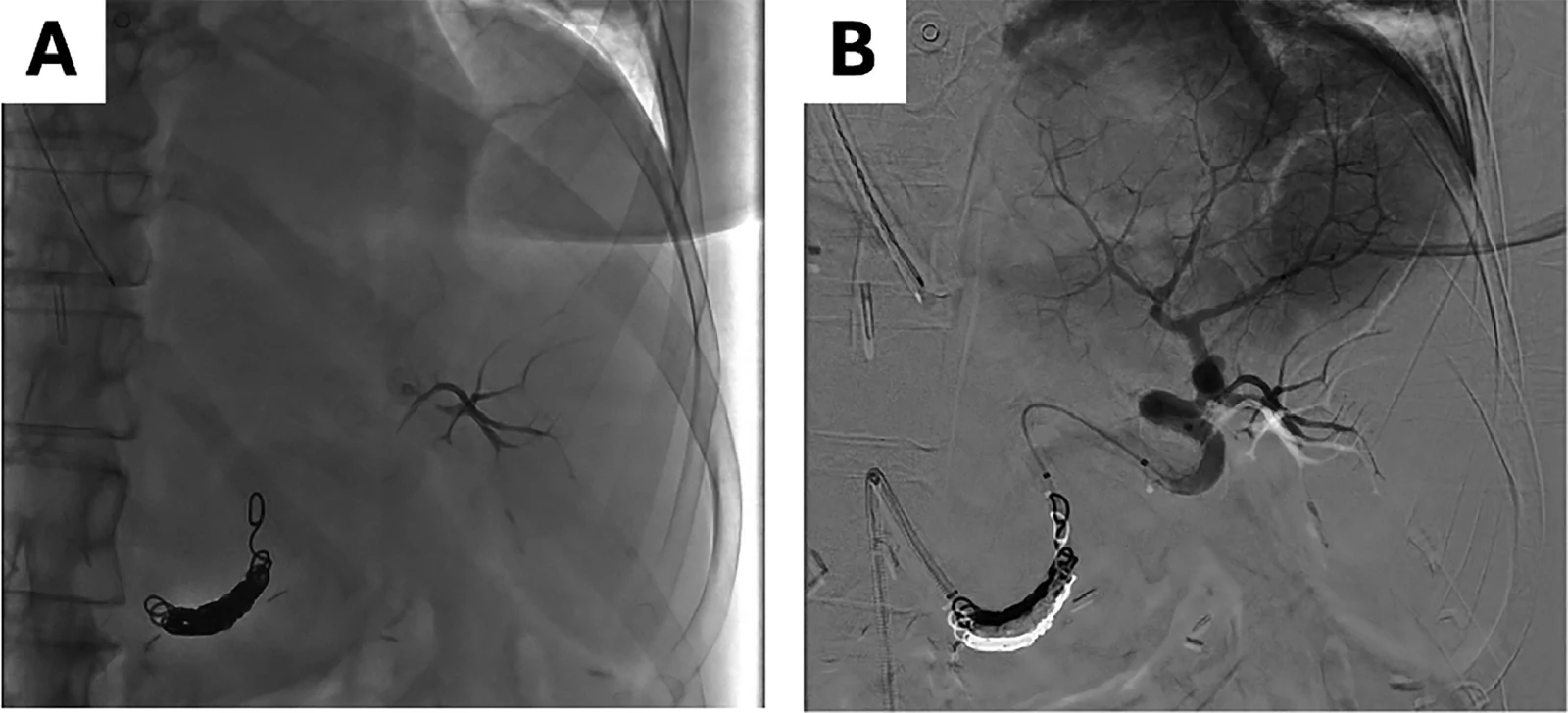
Application of LAVA liquid embolic (A) demonstrating successful distal splenic artery embolization (B).

After informed consent was obtained from the patient, she was placed under general anesthesia. Femoral arterial access was obtained, and a celiac angiogram was performed showing no flow through the previously placed coils, but reconstitution of the distal splenic artery via a complex collateral network from the left gastric artery through the short gastric arteries (Figure 2A) [4]. Attempts to superselect the distal splenic artery through the left gastric collateral network were unsuccessful (Fig. 2B). Prior to accessing the splenic artery percutaneously, recanalization through the coil pack was attempted. A 6Fr × 45 cm Destination sheath (Terumo Interventional Systems, Aliso Viejo, CA) was placed into the celiac artery. A 5Fr CXI catheter (Cook Medical, Bloomington, IN) with a Stiff Angle Glidewire (Terumo Interventional Systems, Aliso Viejo, CA) was then used to recanalize a path through the coil pack (Fig. 3, Fig. 4). Once past the coil pack, tertiary branches of the splenic artery with supply to the inferior pole were embolized with LAVA 18 liquid embolic (SIRTeX, Woburn, MA) (Fig. 5).

## Discussion

This case demonstrates the technical feasibility of crossing a dense coil pack in the proximal splenic artery to permit distal embolization. In this case, a 5 Fr CXI catheter, 6 Fr support sheath, and a 0.035 hydrophilic Glidewire were used to traverse the dense coil pack to re-establish distal splenic artery access. Long-term studies have demonstrated that coil embolization results in durable vessel closure, and to our knowledge, this is the first demonstration of intentional coil-pack recanalization to achieve secondary, distal embolization [5]. This case highlights that, under controlled conditions and appropriate equipment selection, targeted recanalization through a coil pack is an option for intravascular access despite prior proximal coil deployment. While several case reports have demonstrated the safety and feasibility of direct, percutaneous splenic arterial access, this approach increases the risk of hemorrhage and hemoperitoneum, vessel dissection, and pseudoaneurysm [6].

Crossing a dense coil pack is extremely challenging but mechanistically feasible due to the microstructure of a coil pack. The Azure CX series is designed with an inner hydrophilic polymer coating which expands to achieve mechanical vessel occlusion by disrupting flow, but despite expansion, coils do not always obliterate the vessel lumen even when densely packed, especially in patients with coagulapathies [7]. In our case, an adjunctive gelfoam slurry was applied to achieve flow stasis at the time of coil deployment. These coils act as a scaffold for thrombus formation, and by 3 months, thrombus is expected to organize and fibrose around the coils due to fibroblast proliferation and collagen deposition [8].

Thrombus formation is an important consideration in this case as the patient was thrombocytopenic (platelets mean: 24.2, STD: 8.9) during the 2 months between initial coil embolization and subsequent crossing. PT (mean: 13.8, STD: 0.4) and INR (mean: 1.1, STD: 0.1) remained within normal limits. These values suggest a normal coagulation cascade but poor clot formation which would affect thrombus formation within the coil pack. It is very likely the Glidewire did not traverse organized thrombus through the length of the coil pack, and may have instead passed through loosely formed, incomplete clots. Whether crossing a coil pack is feasible after thrombus formation remains unknown and may be possible only prior to clot organization or in coagulopathic patients, and additional studies are warranted.

While this case report demonstrates feasibility, crossing a coil pack carries inherent risks, including coil disruption, migration, or vessel injury. To our knowledge, this is the first reported case of crossing through a remotely placed coil pack to allow distal access and embolization.

## Patient consent

Written informed consent was obtained from the patient for publication of this case report, including all accompanying images and clinical details.
